# Comparison of four glycosyl residue composition methods for effectiveness in detecting sugars from cell walls of dicot and grass tissues

**DOI:** 10.1186/s13068-017-0866-1

**Published:** 2017-07-14

**Authors:** Ajaya K. Biswal, Li Tan, Melani A. Atmodjo, Jaclyn DeMartini, Ivana Gelineo-Albersheim, Kimberly Hunt, Ian M. Black, Sushree S. Mohanty, David Ryno, Charles E. Wyman, Debra Mohnen

**Affiliations:** 1 Department of Biochemistry and Molecular Biology, University of Georgia, Athens, GA 30602 USA; 2 Complex Carbohydrate Research Center, University of Georgia, 315 Riverbend Rd., Athens, GA 30602-4712 USA; 3 DOE-BioEnergy Science Center (BESC), Oak Ridge, 37831 TN USA; 4 Center for Environmental Research and Technology (CE-CERT) and Department of Chemical and Environmental Engineering, University of California Riverside, Riverside, 92507 CA USA; 5 DuPont Industrial Biosciences, Palo Alto, CA 94304 USA; 6 South Georgia State College, Douglas, GA 31533 USA

**Keywords:** Cell wall, Sugar composition, Feedstock, Secondary cell wall, Biofuel, Uronic acid

## Abstract

**Background:**

The effective use of plant biomass for biofuel and bioproduct production requires a comprehensive glycosyl residue composition analysis to understand the different cell wall polysaccharides present in the different biomass sources. Here we compared four methods side-by-side for their ability to measure the neutral and acidic sugar composition of cell walls from herbaceous, grass, and woody model plants and bioenergy feedstocks.

**Results:**

Arabidopsis, *Populus*, rice, and switchgrass leaf cell walls, as well as cell walls from *Populus* wood, rice stems, and switchgrass tillers, were analyzed by (1) gas chromatography–mass spectrometry (GC–MS) of alditol acetates combined with a total uronic acid assay; (2) carbodiimide reduction of uronic acids followed by GC–MS of alditol acetates; (3) GC–MS of trimethylsilyl (TMS) derivatives; and (4) high-pressure, anion-exchange chromatography (HPAEC). All four methods gave comparable abundance ranking of the seven neutral sugars, and three of the methods were able to quantify unique acidic sugars. The TMS, HPAEC, and carbodiimide methods provided comparable quantitative results for the specific neutral and acidic sugar content of the biomass, with the TMS method providing slightly greater yield of specific acidic sugars and high total sugar yields. The alditol acetate method, while providing comparable information on the major neutral sugars, did not provide the requisite quantitative information on the specific acidic sugars in plant biomass. Thus, the alditol acetate method is the least informative of the four methods.

**Conclusions:**

This work provides a side-by-side comparison of the efficacy of four different established glycosyl residue composition analysis methods in the analysis of the glycosyl residue composition of cell walls from both dicot (Arabidopsis and *Populus*) and grass (rice and switchgrass) species. Both primary wall-enriched leaf tissues and secondary wall-enriched wood/stem tissues were analyzed for mol% and mass yield of the non-cellulosic sugars. The TMS, HPAEC, and carbodiimide methods were shown to provide comparable quantitative data on the nine neutral and acidic sugars present in all plant cell walls.

**Electronic supplementary material:**

The online version of this article (doi:10.1186/s13068-017-0866-1) contains supplementary material, which is available to authorized users.

## Background

Cell walls constitute the bulk of plant biomass, a major renewable resource for biofuel and biomaterial production [1, 2]. The use of plants as a source for bioproducts [3] is expected to increase as fossil fuel supplies decrease, mitigation strategies for climate change intensify [4], and the world population increases [5]. Plant cell walls are a matrix of complex non-cellulosic polymers (hemicelluloses and pectins), cellulose microfibrils and fibers, proteins and proteoglycans, and in tissues with secondary walls, the phenolic polymer lignin [6]. The choice of plant species for production of specific bioproducts is influenced by the quality and quantity of the cell wall polymers [7]. Although all plant cell walls contain the same general types of polymers, the specific amounts of the different polymers and their unique glycosyl residue content and linkages vary in different types of plants (e.g., woody versus herbaceous dicots versus grasses) and in different tissues and cell types. Since the different cell wall polymers have unique physical–chemical properties, their suitability as a resource for specific bioproducts also varies, underscoring the need for cell wall analysis methods that can detect critical differences in different biomass sources.

A full analysis of cell wall structure requires the use of detailed, time-consuming, and often expensive analytical methods [8]. However, an initial assessment of the polymer content of biomass samples can be obtained by analysis of the glycosyl residue composition. Multiple methods exist to measure the sugar content of plant cell walls; however, these methods have not been compared side-by-side for effectiveness in analyzing the same tissues from multiple plant species. Here we compare the four most common sugar composition methods for their ability to reproducibly quantify the greatest number of different types of sugars present in cell walls of dicot and grass species. The goal was to provide researchers a reference source for selecting a preferred sugar analysis method for comparison of cell walls from different species, cell types, and/or walls from native versus mutant/transgenic/variant plants.

**Fig. 1:**
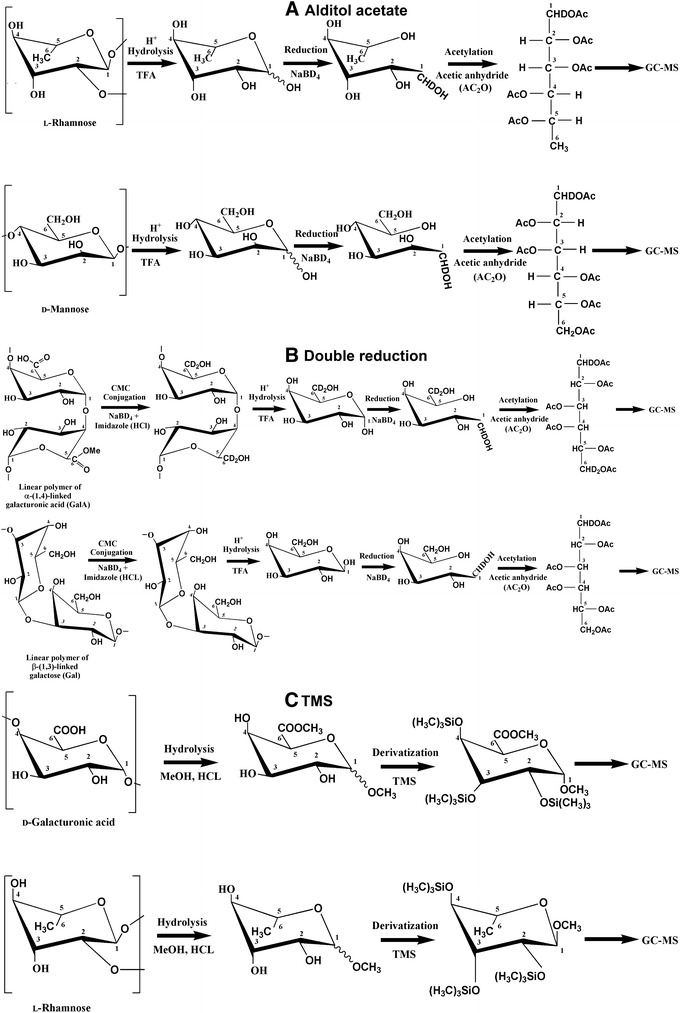
Schematic overview of the derivatization of sugar residues by the **A** alditol acetate, **B** carbodiimide, and **C** TMS plant cell wall glycosyl residue composition analysis methods. Schematic depicts analysis of the designated terminal and internal sugars of the indicated plant cell wall polysaccharides: **A** terminal Rha and internal Man residues, **B** internal GalA residues of homogalacturonan and Gal residues in β-1,3-linked galactan, **C** internal GalA and terminal Rha residues. Cyclic and linear sugars are depicted as Haworth and Fischer projections, respectively

The two most common plant cell wall sugar composition analysis methods are the alditol acetate (AA) and the trimethylsilyl (TMS) methods [9]. The AA method involves hydrolysis of monosaccharides from alcohol insoluble residues (AIR) and their reduction to alditols using sodium borohydride, followed by acetylation with acetic anhydride to volatilize them for gas chromatography and mass spectrometry (GC–MS) (Fig. 1A) [10]. The AA method has been used to study the sugar composition of many plant species, including Arabidopsis [11], Italian ryegrass [10], potatoes [12], barley [13], tobacco [14], *Populus* [15], rice [16], and switchgrass [17]. The limitation of this method is that it does not measure acidic sugars. In contrast, the TMS method involves sequential methanolysis and trimethylsilylation of the hydrolyzed sugars to yield TMS-methyl glycosides (Fig. 1C), enabling detection of both neutral and acidic sugars. The TMS method has been used to study sugar composition in a variety of plant species including Arabidopsis [18], rice [19], carrots and apples [20], and *Populus* [21].

Uronic acids (UAs) are ubiquitous acidic sugars in plant cell wall non-cellulosic polysaccharides, including galacturonic acid (GalA) in pectins and glucuronic acid (GlcA) in hemicellulosic xylans. UAs are abundant in dicot primary walls, of lesser abundance in dicot secondary walls, and of low abundance in grass walls. Many researchers have thus used the AA method to analyze the cell walls of grasses and dicot secondary walls. However, this results in an underestimation, or total lack of recognition, of the presence of UAs in such biomass, as well as the risk of not identifying UA-containing matrix polysaccharides, such as pectin and glucuronoxylan that have been shown to impact biomass recalcitrance [15, 21–23]. Thus, the glycosyl residue composition analysis methods that detect both neutral and acidic sugars, such as the TMS method, are preferable for the most complete analyses. However, despite its advantage over the AA method, the TMS method is not without drawbacks. TMS derivatization of methyl glycosides results in multiple anomeric forms of the monosaccharide derivatives, yielding multiple peaks for each sugar that can be difficult to distinguish and quantitate [20, 24].

Another method to analyze both neutral and acidic sugars is the carbodiimide method, which entails reduction of UAs to their respective neutral sugars with subsequent analysis by the AA method (Fig. 1B) [25]. Specifically, the carboxyl groups of UAs in un-degraded polymeric material are activated with a water-soluble carbodiimide and reduced with sodium borodeuteride to yield 6,6-dideuterio sugars. The UAs are quantified as the increased amount of their respective neutral sugars in a pre-reduced compared to un-reduced sample. This method has been used to study the cell wall sugar composition of apple [26] and maize [25].

Liquid chromatography-based methods are also available that detect both neutral and acidic sugars in hydrolyzed cell wall samples. High-pressure, anion-exchange chromatography (HPAEC) coupled with electrochemical detection (ECD) allows for direct analysis of monosaccharides and oligosaccharides without derivatization or labeling. It uses high pH (pH 12–13) to partially deprotonate the sugar hydroxyl groups, yielding sugar anions that can be separated on anion-exchange columns [27, 28]. This method has been used to analyze cell walls from multiple plant species including Arabidopsis [29], wheat [30], potato [31], rice [32], and switchgrass [17]. HPAEC, however, has the disadvantage that it is not readily adaptable to mass spectrometry for confirmation of sugar identity.

Here we compare four different sugar composition analysis methods (AA, carbodiimide, TMS, and HPAEC) for their ability to quantify the sugar composition of cell walls from leaves of Arabidopsis, *Populus*, rice, and switchgrass. Our objective was to identify quantitative, reliable, and facile methods for analysis of the glycosyl residue composition of plant cell walls. Such information is essential to understand plant cell wall structure/function relationships and cell wall structures associated with biomass recalcitrance and/or bioproduct quality. To the best of our knowledge, this is the first side-by-side comparison of the different analytical methods using the same tissue sources from multiple dicot and grass species. To ensure that the results from the analysis of leaves are applicable to other types of biomass tissues (e.g., stems), we also compared the performance of the four methods in the analysis of cell walls from *Populus* wood, rice stem, and switchgrass tillers. We conclude that the TMS, HPAEC, and carbodiimide methods are the preferred methods to obtain quantitative and reproducible sugar composition data on the major neutral and acidic sugars present in all dicot and grass biomass.

## Methods

### Plant material and growth conditions

Arabidopsis wild-type (WT) [*Arabidopsis thaliana* (L.) Heynh. var. Columbia S6000] plants were grown essentially as described [33]. Briefly, sterilized seeds were sown on media plates containing half-strength Murashige and Skoog basal salts (Sigma-Aldrich Corp., St. Louis, MO) and 5.5 g/L plant agar (Research Products International Corp., Mount Prospect, IL) with pH adjusted to 5.7 prior to autoclaving. Seed-containing plates were kept in a growth chamber with 60% relative humidity, 150 μmol photons/m^2^/s light, and photoperiod cycle of light for 14 h at 19 °C and dark for 10 h at 15 °C. Following germination, 10-day-old seedlings were transferred to soil and grown to maturity in a growth chamber under the same growth conditions as above. Fertilizer (Peters 20/20/20 with micronutrients) was applied once a week or as needed.

*Populus deltoides* Bartr. ex Marsh. clone WV94 plants were obtained from ArborGen Inc. (Ridgeville, SC) as plantlets generated in vitro from petiole explants via callus. Rooted plantlets grown for 4–6 weeks in magenta boxes were cleaned with running water to remove media and charcoal, and transplanted into soil in 3.8-L (1 gallon) pots. Growth conditions and plant maintenance were as previously described [21] and summarized below. The soil was a mix of 1 bag (2.8 cubic feet) of Fafards 3B Soil mix (GroSouth Inc, Atlanta, GA), 250 mL osmocote, 84 mL bone meal, 84 mL gypsum, and 42 mL dolomite/limestone. Plants were grown in the greenhouse for 9 months under a 16-h light/8 h dark cycle at 25–32 °C with constant misting, and fertilized weekly with Peters 20-10-20 **(**nitrogen-phosphorus-potassium; GroSouth Inc, Atlanta, GA).

Switchgrass (*Panicum virgatum* L.) var. Alamo II genotype ST1 [34] was grown for 2 months as seedlings in 3.8-L (1 gallon) pots followed by transfer to 11.4-L (3 gallon) pots and a further 6 weeks of growth. Growing medium was a soil mixture consisting of two 2.8-cubic-feet bags of Fafards 3B (GroSouth Inc, Atlanta, GA), one 2.8-cubic-feet of River Bottom Sand (Redland Sand, Watkinsville, GA), and 118 mL of Osmocote Plus granular fertilizer (18-9-12 minors, 8–9 month release). After planting, plants were fertilized once a week with 440 ppm Jack’s Peat Lite Special 20-10-20 (nitrogen-phosphorus-potassium; GroSouth Inc, Atlanta, GA).

Rice (*Oryza sativa* L.) seeds var. IAC 165 obtained from the USDA National Plant Germplasm System were grown in 1.9-L (1/2 gallon) pots for 2 weeks. The seedlings were then transferred to 11.4-L (3 gallon) pots and grown in a greenhouse under a 16-h light/8 h dark cycle at 25–32 °C. Growing medium was the same soil mixture as described above for switchgrass. At the time of planting, plants were fertilized with 1.2-mL (1/4 teaspoon) Sprint 330 Iron Chelate and 3.75 g Jack’s Peat Lite Special 20-10-20 per 2 L water. After planting, plants were fertilized once a week with 440 ppm of the Peat Lite Special 20-10-20.

### Isolation of plant samples and preparation of cell walls as alcohol insoluble residues (AIR)

Leaf samples were harvested from 5-week-old Arabidopsis, 8-week-old rice, and 10-week-old switchgrass and *Populus*, ground to a fine powder using liquid nitrogen, and stored at −80 °C until use. Biomass samples were isolated as follows: rice stem from 3-month-old plants, switchgrass whole tillers harvested at the R1 stage [35], and *Populus* wood from 9-month-old plants [21]. Harvested biomass samples were air dried completely and milled to a 20-mesh (0.85 mm) particle size using a Wiley Mini-Mill (model number: 3383L10, Thomas Scientific). For *Populus* wood, the bottom 6 cm of stem measured from the soil surface was collected from 9-month-old plants, the bark peeled using a razor, the remaining stem air dried, and the pith removed using a hand drill prior to milling. AIR was prepared from the ground tissue/biomass powder and de-starched prior to analysis as described [33].

### Glycosyl residue composition analysis by the alditol acetate (AA) derivatization method

The neutral sugar composition of AIR was analyzed by the AA derivatization method [36] with modification. Briefly, 100–500 µg AIR was incubated in 0.2–1 mL 2 M trifluoroacetic acid (TFA, Thermo Fisher Scientific, Waltham, MA) at 121 °C for 2 h, followed by reduction with 200–300 μL of 10 mg/mL NaBD_4_ in 1 M ammonium hydroxide for at least 2 h to overnight at room temperature (RT). The borodeuteride solution was neutralized by adding 3–4 drops glacial acetic acid, and dried down twice with 200 μL methanol:acetic acid (9:1 [v/v]) and thrice with 200 μL anhydrous methanol under a stream of air. The samples were incubated with 250 μL acetic anhydride and 250 μL concentrated TFA for 10 min at 50 °C and dried down with 20–30 drops of isopropanol under a stream of air. To the dried samples, 1 mL of 0.2 M sodium carbonate and 1 mL of methylene chloride (Sigma) was added, the samples vortexed, and the upper aqueous layer removed. The bottom organic layer containing AA derivatives of hydrolyzed sugars was washed thrice with 1 mL deionized water (ddH_2_O), transferred to a clean tube, dried down, and resuspended in ~100 μL methylene chloride. The samples (~1 μL) were injected using the splitless injection mode and helium as carrier gas onto an SP-2330 Supelco column (30 m × 0.25 mm, 0.25 μm film thickness) connected to a Hewlett–Packard chromatograph (5890) coupled to a mass spectrometer for GC–MS analysis. AA derivatives were separated using the following temperature gradient: 80 °C for 2 min, 80–170 °C at 30 °C/min, 170–240 °C at 4 °C/min, and 240 °C for 20 min, and were ionized by electron impact at 70 eV. A sample of the GC profile of alditol acetate derivatives of the neutral sugar standards is provided in Additional file 1. GC peak areas were used to determine the response factors for each sugar relative to the internal standard myo-inositol, and subsequently used to determine the amount of sugars in the wall samples [8].

### Colorimetric determination of uronic acid (UA) content

The UA content of AIR samples was determined using a modification of the methods of Blumenkrantz and Asboe-Hansen [37], Filisetti-Cozzi and Carpita [38], and van den Hoogen et al. [39] as described below. The hydrolysis of AIR samples was performed independently with either sulfuric acid (H_2_SO_4_) or TFA [40]. Briefly, 0.4 mg AIR was suspended in 0.4 mL ddH_2_O and mixed thoroughly with 40 μL of 4 M sulfamic acid–potassium sulfamate (pH 1.6). The sample was subsequently hydrolyzed with 2.4 mL of 12.5 mM sodium tetraborate in either concentrated H_2_SO_4_ or 2 M TFA, with incubation for 20 min at 100 °C for H_2_SO_4_ hydrolysis or for 2 h at 120 °C for TFA hydrolysis. The reaction mixture was cooled immediately, and mixed with 80 μL of 0.15% (w/v) *m*-hydroxybiphenyl in 0.5% (w/v) NaOH by vortexing. After 5–10 min, the pink color that developed was measured as absorbance at 540 nm using a microtiter plate reader and the UA content was estimated by comparison to a standard curve of GalA (Sigma-Aldrich, St. Louis, MO) as illustrated in Additional file 2.

### Glycosyl residue composition analysis by uronic acid reduction using the carbodiimide method

Glycosyl residue composition was analyzed by initial activation of UAs in an underivatized sample using the carbodiimide method [41] followed by reduction with NaBD_4_ to convert UAs to their respective 6,6-dideuterio sugars [25, 42, 43]. The AIR sample (10 mg) was suspended in 3 mL 0.033 M sodium acetate pH 4.6. With continuous stirring, 250 mg of CMC [*N*-cyclohexyl-*N’*-(2-morpholinoethyl) carbodiimide] methyl-*p*-toluene sulfonate (Sigma) powder was added, and the pH kept at 4.8 by dropwise addition of 1 M HCl for 2 h. The mixture was chilled on ice, mixed with 1 mL ice cold 4 M imidazole–HCl pH 7.0, and immediately 300 mg NaBD_4_ was added to the suspension with continuous stirring on ice for 1 h. Excess borodeuteride was afterwards destroyed by dropwise addition of glacial acetic acid. The sample was dialyzed against running ddH_2_O for at least 36 h, frozen, lyophilized [26], and one mg of the lyophilized material subjected to sugar composition analysis by the AA method as described above. The amount of the UAs, GalA, and GlcA was calculated as the increase in the amount of galactose (Gal) and glucose (Glc), respectively, compared to the amount measured in un-reduced samples analyzed directly using the AA method [42].

### Glycosyl residue composition analysis by GC–MS of TMS-derivatized methyl glycosides

Glycosyl residue composition of AIR was determined by GC–MS of per-*O*-trimethylsilyl (TMS) derivatives of monosaccharide methyl glycosides produced by acidic methanolysis as previously described [8, 44]. AIR (100–300 µg) was aliquoted into individual tubes, supplemented with 20 μg inositol as internal standard, and lyophilized. The dry samples were hydrolyzed for 18 h at 80 °C in 1 M methanolic-HCl (Supelco, St. Louis, MO), cooled to RT, evaporated under a stream of air, and dried twice more with anhydrous methanol. The released glycosyl residues were derivatized with 200 μL TriSil Reagent (Thermo Fisher Scientific, Waltham, MA) at 80 °C for 20 min. Cooled samples were evaporated under a stream of air, resuspended in 3 mL hexane, and filtered through packed glass wool. Dried samples were resuspended in 150 μL hexane and 1 μL sample injected using helium as carrier gas onto a Supelco EC-1 fused silica capillary column (30 m × 0.25 mm ID) on an Agilent 7890A gas chromatograph interfaced to a 5975C mass spectrometer. The temperature gradient was: 80 °C for 2 min, 80–140 °C at 20 °C/min, 140–200 °C at 2 °C/min, and 200–250 °C at 30 °C/min. A sample of the GC profile of the TMS-derivatized sugar standards is provided in Additional file 3. The response factor for each sugar was determined from the GC peak area (or total peak areas of multiple peaks if multiple derivatives were formed) of each sugar standard relative to the internal standard myo-inositol, and the value subsequently used to calculate the amount of each sugar in the wall samples [8].

### Glycosyl residue composition analysis by HPAEC

AIR (100 µg) was refluxed in 400 µL 2 M TFA at 120 °C for 1 h [31, 45] and the resulting solution dried under a stream of air with addition of isopropanol. The dried residue was dissolved in 200 µL ddH_2_O and the solution centrifuged for 5 min. The supernatant was diluted 1:3 with ddH_2_O and 50 µL of the diluted supernatant injected into a Dionex ICS-3000 HPLC system (Dionex, Sunnyvale, CA) for monosaccharide analysis by high pH anion-exchange chromatography with electrochemical detection in the carbohydrate mode. The buffers used were A—nanopure water, B—200 mM NaOH, and C—1 M NaOAc. Two programs were used to detect different monosaccharides. Program 1 was used to quantify fucose (Fuc), rhamnose (Rha), arabinose (Ara), Gal, Glc, GalA, and GlcA using a Dionex PA20 column (3 × 150 mm) at a 0.5 mL/min flow rate (see Additional file 4A for a sample chromatogram). The column was equilibrated at 1% buffer B for 30 min prior to each separation. The gradient was: 0 min 1% buffer B, 0.1 min 10% buffer B, 2 min 10% buffer B, 4 min 1% buffer B, 15 min 0% buffer B, 25 min 5% buffer B and 10% buffer C, 30 min 5% buffer B and 50% buffer C, and 35 min 1% buffer B. Program 2 was used to quantify xylose (Xyl) and mannose (Man) (see Additional file 4B) using a Dionex PA1 column (4 × 150 mm) and a flow rate of 1 mL/min. The column was equilibrated with 1% of buffer B for 30 min, the sample eluted isocratically at 1% buffer B for 40 min, and the column regenerated with 100% buffer B for 5 min. For both programs, a standard mixture containing known concentrations of different sugars was used to plot concentration-peak area standard curves. The amount of each monosaccharide was calculated from the standard equations based on the corresponding peak area as registered by ECD.

## Results

Our goal was to determine the preferred sugar composition analysis method(s) for use across dicot and monocot grass species, which have been shown to have characteristically distinct cell wall compositions and structures [46–48]. *Populus* and switchgrass were chosen as bioenergy crops and Arabidopsis and rice as model plants for the comparison. Leaves were used as the initial target tissue for this study for three reasons. First, leaves are comparable organs between these two groups. In contrast, for example, *Populus* wood has a different tissue structure and composition compared to rice and switchgrass tillers. Secondly, leaves are a major biomass resource. For example, leaves comprise a significant proportion (25–44%) of switchgrass biomass [49]. Thirdly, leaves are composed of both primary and secondary walls, and thus contain the majority of the different types of cell wall polysaccharides.

**Fig. 2:**
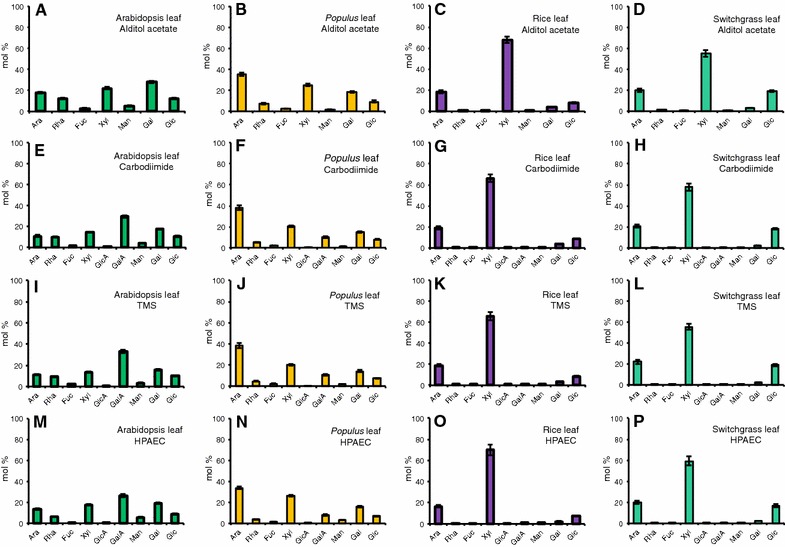
Comparison of the glycosyl residue composition of leaf AIR from cell walls of Arabidopsis, Populus, rice, and switchgrass obtained by **A**–**D** GC–MS of alditol acetate derivatives; **E**–**H** the carbodiimide method; **I**–**L** GC–MS of TMS (trimethylsilane) derivatives; and **M**–**P** the HPAEC method. Data are average mol% monosaccharide quantified from two technical replicates of each of three biological replicates ± standard deviation, *n* = 6 [exceptions are three technical replicates for uronic acid assay (*n* = 9) and a single technical replicate for HPAEC method (*n* = 3)]. Monosaccharide abbreviations: arabinose (Ara), rhamnose (Rha), fucose (Fuc), xylose (Xyl), galacturonic acid (GalA), glucuronic acids (GlcA), mannose (Man), galactose (Gal), and glucose (Glc)

**Fig. 3:**
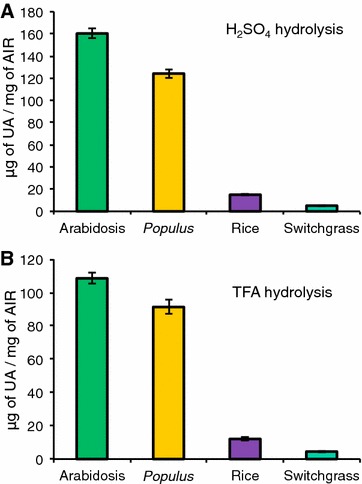
Determination of uronic acid content of AIR from leaves of Arabidopsis, *Populus*, rice, and switchgrass. Uronic acid assays were carried out using either **A** H_2_SO_4_ or **B** TFA hydrolysis. Data are means of three technical replicates of each of three biological replicates ± standard deviation, *n* = 9

**Fig. 4:**
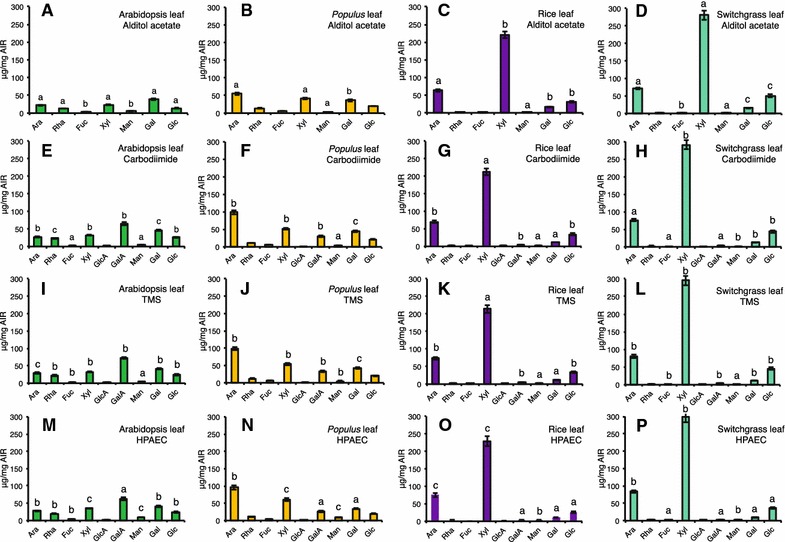
Comparison of the glycosyl residue composition of leaf AIR from cell walls of Arabidopsis, *Populus*, rice, and switchgrass obtained by **A**–**D** GC–MS of alditol acetate derivatives; **E**–**H** the carbodiimide method; **I**–**L** GC–MS of TMS (trimethylsilane) derivatives; and **M**–**P** the HPAEC method. Data are average µg monosaccharide quantified per mg of leaf AIR from two technical replicates of each of three biological replicates ± standard deviation, *n* = 6 [exceptions are three technical replicates for uronic acid assay (*n* = 9) and a single technical replicate for HPAEC method (*n* = 3)]. Monosaccharide abbreviations: arabinose (Ara), rhamnose (Rha), fucose (Fuc), xylose (Xyl), galacturonic acid (GalA), glucuronic acids (GlcA), mannose (Man), galactose (Gal), and glucose (Glc). *Different letters* indicate significant differences between the amounts (“a” represents the lowest amount) of a particular sugar residue in the cell walls of a species across different methods (and not between different types of sugars within that biomass sample analyzed using a particular method). Statistics are one-way analysis of variance (ANOVA) followed by Tukey’s multiple comparison tests with significant *P* value <0.05

**Fig. 5:**
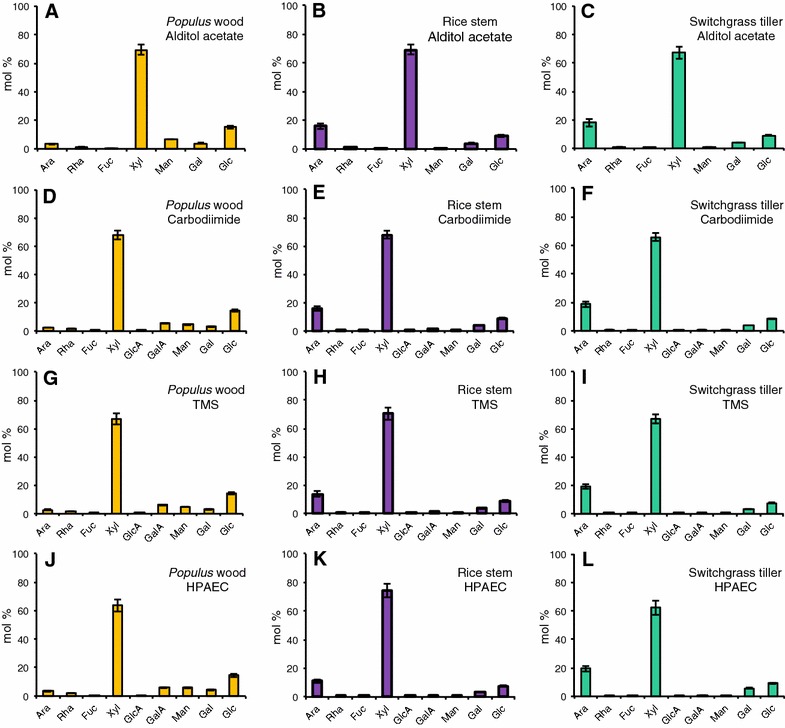
Comparison of the glycosyl residue composition of AIR from cell walls of Populus wood, rice stem, and switchgrass tiller biomass obtained by **A**–**C** GC–MS of alditol acetate derivatives; **D**–**F** the carbodiimide method; **G**–**I** GC–MS of TMS (trimethylsilane) derivatives; and **J**–**L** the HPAEC method. Data are average mol% monosaccharide quantified from two technical replicates of each of three biological replicates ± standard deviation, *n* = 6 [exceptions are three technical replicates for uronic acid assay (*n* = 9) and a single technical replicate for HPAEC method (*n* = 3)]. Monosaccharide abbreviations: arabinose (Ara), rhamnose (Rha), fucose (Fuc), xylose (Xyl), galacturonic acid (GalA), glucuronic acids (GlcA), mannose (Man), galactose (Gal), and glucose (Glc)

**Fig. 6:**
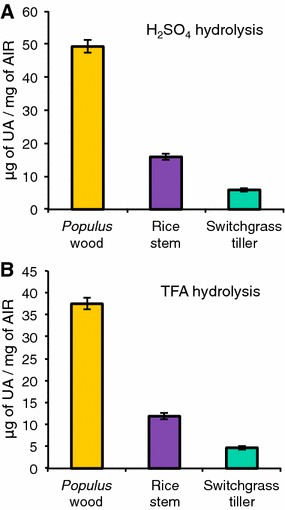
Determination of uronic acid content of AIR from *Populus* wood, rice stem, and switchgrass tiller biomass. Uronic acid assays were carried out using either **A** H_2_SO_4_ or **B** TFA hydrolysis. Data are the means of three technical replicates of each of three biological replicates ± standard deviation, *n* = 9

**Fig. 7:**
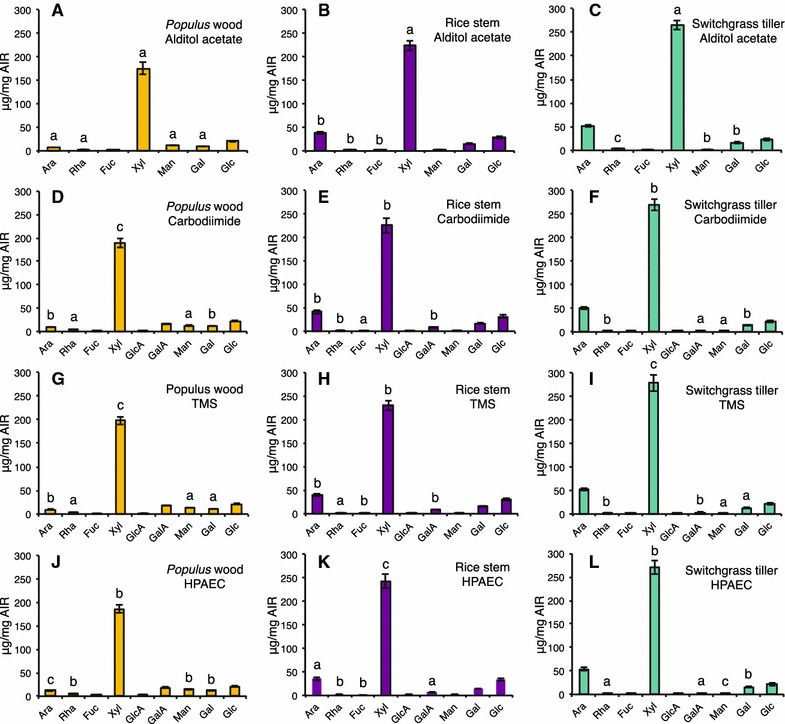
Comparison of the glycosyl residue composition of biomass AIR from cell walls of Populus wood, rice stem, and switchgrass tiller obtained by **A**–**C** GC–MS of alditol acetate derivatives; **D**–**F** the carbodiimide method; **G**–**I** GC–MS of TMS (trimethylsilane) derivatives; and **J**–**L** the HPAEC method. Data are average μg monosaccharide quantified per mg of leaf AIR from two technical replicates of each of three biological replicates ± standard deviation, *n* = 6 [exceptions are three technical replicates for uronic acid assay (*n* = 9) and a single technical replicate for HPAEC method (*n* = 3)]. Monosaccharide abbreviations: arabinose (Ara), rhamnose (Rha), fucose (Fuc), xylose (Xyl), galacturonic acid (GalA), glucuronic acids (GlcA), mannose (Man), galactose (Gal), and glucose (Glc). *Different letters* indicate significant differences between the amounts (“a” represents the lowest amount) of a particular sugar residue in the cell walls of a species across different methods (and not between different types of sugars within that biomass sample analyzed using a particular method). Statistics are one-way analysis of variance (ANOVA) followed by Tukey’s multiple comparison tests with significant *P* value <0.05

We first compared the four different glycosyl residue composition analysis methods for their ability to measure the nine major neutral and acidic sugars in leaf AIR from Arabidopsis, *Populus*, rice, and switchgrass. We present the data in both the relative yield (mol%) (Fig. 2; Additional file 5) and the mass yield (μg sugar/mg AIR) (Table 1; Figs. 3, 4). Mol% data provide information on the relative molar proportions of the different sugar residues, which are indicative of the relative amounts of different non-cellulosic wall polymers in different wall samples. As such, mol% data provide a facile means to compare sugar compositions of different cell wall samples, even when the total amount of polysaccharides in the walls differs as can be the case, for example, in mutant versus wild-type samples [50]. Mass yield data provide information on the actual measurable amounts of the different sugars present in cell walls from different plant samples, and thus, are indicative of the effectiveness of the methods in quantifying both major and minor sugars. Finally, we further compared the efficacy of the four methods in the analysis of AIR from *Populus* wood, rice stem, and switchgrass tiller, which represent biomass from secondary cell-wall-enriched biofuel feedstock tissues (Table 2; Figs. 5, 6, 7; Additional file 6).

**Table 1:** Comparison of different glycosyl residue analysis methods for μg monosaccharide quantified per mg of AIR from four different leaf samples

Leaf-method	μg glycosyl residue/mg leaf AIR											
	Ara	Rha	Fuc	Xyl	GlcA	GalA	Man	Gal	Glc	Total neutral	Total acidic	Total
Arabidopsis												
Alditol acetate	21.8 ± 0.8	13.3 ± 0.7	3.5 ± 0.3	23.9 ± 1.3	–	–	5.2 ± 0.3	38.6 ± 2.3	14.0 ± 0.6	120.3		120.3
Uronic acid*	–	–	–	–	108.6 ± 3.2		–	–	–		108.6	
Alditol acetate + uronic acid**	21.8 ± 0.8^a^	13.3 ± 0.7^a^	3.5 ± 0.3^b^	23.9 ± 1.3^a^	108.6 ± 3.2		5.2 ± 0.3^b^	38.6 ± 2.3^a^	14.0 ± 0.6^a^	120.3^a^	108.6^c^	228.9^a^
Carbodiimide	27.4 ± 1.8^b^	23.5 ± 1.5^c^	2.7 ± 0.02^a^	32.7 ± 1.4^b^	1.7 ± 0.01	64.8 ± 4.1^b^	4.8 ± 0.3^a^	46.6 ± 2.1^c^	26.1 ± 1.3^b^	163.8^b^	66.5^a^	230.2^b^
TMS	29.2 ± 2.2^c^	22.1 ± 1.9^b^	3.5 ± 0.03^b^	32.2 ± 1.1^b^	1.9 ± 0.02	72.5 ± 3.3^b^	5.2 ± 0.5^a^	42.3 ± 2.6^b^	24.3 ± 2.2^b^	158.8^b^	74.4^b^	233.1^b^
HPAEC	27.4 ± 1.4^b^	20.0 ± 1.0^b^	3.3 ± 0.02^b^	35.5 ± 1.5^c^	1.8 ± 0.01	62.4 ± 3.9^a^	8.9 ± 0.7^c^	41.0 ± 2.8^b^	23.4 ± 1.6^b^	159.5^b^	64.2^a^	223.6^a^
*Populus*												
Alditol acetate	55.2 ± 3.8	13.6 ± 0.7	5.3 ± 0.2	41.5 ± 2.6	–	–	3.9 ± 0.4	35.7 ± 2.5	20.5 ± 0.8	175.7		175.7
Uronic acid*	–	–	–	–	91.4 ± 4.1		–	–	–		91.4	
Alditol acetate + uronic acid**	55.2 ± 3.8^a^	13.6 ± 0.7	5.36 ± 0.2	41.5 ± 2.6^a^	91.4 ± 4.1		3.9 ± 0.4^a^	35.7 ± 2.5^b^	20.5 ± 0.8	175.7^a^	91.4^c^	267.1^a^
Carbodiimide	98.1 ± 5.1^b^	11.2 ± 0.7	5.1 ± 0.2	51.5 ± 2.9^b^	0.9 ± 0.01	29.5 ± 3.1^b^	3.6 ± 0.3^a^	44.6 ± 2.9^c^	21.2 ± 1.3	235.3^b^	30.4^a^	265.7^a^
TMS	99.2 ± 4.9^b^	12.4 ± 0.9	5.9 ± 0.4	54.1 ± 3.2^b^	1.0 ± 0.01	32.8 ± 2.9^b^	5.1 ± 0.4^b^	42.3 ± 2.1^c^	20.2 ± 1.8	239.2^b^	33.8^b^	272.8^b^
HPAEC	95.7 ± 6.3^b^	10.6 ± 0.8	4.2 ± 0.3	60.3 ± 4.1^c^	0.7 ± 0.01	25.6 ± 2.6^a^	8.6 ± 0.6^c^	34.3 ± 1.8^a^	19.7 ± 1.4	233.4^b^	26.3^a^	259.7^a^
Rice												
Alditol acetate	64.1 ± 3.9	2.8 ± 0.1	0.7 ± 0.02	220.3 ± 8.9	-	-	1.3 ± 0.01	16.5 ± 1.1	31.1 ± 2.4	336.8		336.8
Uronic acid*	-	-	-	-	12.1 ± 0.9		-	-	-		12.1	
Alditol acetate + uronic acid**	64.1 ± 3.9^a^	2.8 ± 0.1	0.7 ± 0.02	220.3 ± 8.9^b^	12.1 ± 0.9		1.3 ± 0.01^a^	16.5 ± 1.1^b^	31.1 ± 2.4^b^	336.8^a^	12.1^c^	348.9^c^
Carbodiimide	70.3 ± 3.6^b^	3.1 ± 0.3	0.5 ± 0.01	212.3 ± 9.3^a^	1.1 ± 0.20	4.3 ± 0.4^b^	1.2 ± 0.02^a^	11.6 ± 0.8^a^	34.3 ± 2.8^b^	333.3^a^	5.4^a^	338.5^a^
TMS	73.4 ± 4.2^b^	2.8 ± 0.3	0.4 ± 0.01	214.5 ± 11.3^a^	1.2 ± 0.02	4.9 ± 0.4^b^	2.0 ± 0.01^a^	11.4 ± 0.9^a^	33.5 ± 1.9^b^	338.0^a^	6.1^b^	343.9^b^
HPAEC	75.3 ± 5.8^c^	2.9 ± 0.2	0.8 ± 0.02	228.9 ± 14.3^c^	0.6 ± 0.01	3.6 ± 0.3^a^	3.1 ± 0.04^b^	10.8 ± 0.6^a^	25.6 ± 2.1^a^	347.2^b^	4.4^a^	351.6^c^
Switchgrass												
Alditol acetate	71.3 ± 2.4	2.5 ± 0.3	1.0 ± 0.02	281.0 ± 13.3	-	-	1.4 ± 0.02	15.8 ± 1.6	50.2 ± 4.1	423.2		423.2
Uronic acid*	-	-	-	-	4.5 ± 0.4		-	-	-		4.5	
Alditol acetate + uronic acid**	71.3 ± 2.4^a^	2.5 ± 0.3	1.0 ± 0.02^b^	281.0 ± 13.3^a^	4.5 ± 0.4		1.4 ± 0.02^a^	15.8 ± 1.6^c^	50.2 ± 4.1^c^	423.2^a^	4.5	427.7^a^
Carbodiimide	75.8 ± 3.3^a^	2.5 ± 0.3	0.6 ± 0.01^a^	290.8 ± 12.7^b^	0.8 ± 0.01	3.6 ± 0.2^a^	2.0 ± 0.02^b^	12.6 ± 0.7^b^	43.7 ± 3.9^b^	428.0^a^	4.4	432.3^a^
TMS	80.3 ± 5.1^b^	2.7 ± 0.4	0.8 ± 0.01^b^	295.3 ± ± 13.8^b^	0.9 ± 0.01	4.1 ± 0.4^b^	1.0 ± 0.01^a^	11.9 ± 1.0^b^	45.3 ± 3.5^b^	437.3^b^	5.0	442.3^b^
HPAEC	84.0 ± ± 3.7^b^	2.0 ± 0.2	0.6 ± 0.01^a^	299.3 ± 15.8^b^	0.9 ± 0.01	3.0 ± 0.3^a^	2.3 ± 0.02^b^	9.0 ± 0.9^a^	36.0 ± 2.8^a^	433.2^b^	3.9	437.1^b^

Data are means of three biological replicates ± standard deviation, with two technical replicates for alditol acetate, carbodiimide, and TMS methods (*n* = 6), three technical replicates for uronic acid assay (*n* = 9), and a single technical replicate for HPAEC method (*n* = 3). Different letters in superscript following the values indicate significant differences between the amounts (“a” represents the lowest amount) of a particular (or total) sugar residue in the cell walls of a species across different methods (and not between different types of sugars within that leaf sample analyzed using a particular method). Statistics are one-way analysis of variance (ANOVA) followed by Tukey’s multiple comparison tests with significant *P* value <0.05

* Uronic acid assay data shown here are from analysis using TFA hydrolysis

** Alditol acetate + uronic acid data shown here are the combined data of the respective individual assays

**Table 2:** Comparison of different glycosyl residue analysis methods for μg monosaccharide quantified per mg of AIR from three different biofuel feedstock biomass samples from three different species

Biomass-method	μg glycosyl residue/mg biomass AIR											
	Ara	Rha	Fuc	Xyl	GlcA	GalA	Man	Gal	Glc	Total neutral	Total acidic	Total
*Populus* wood												
Alditol acetate	6.7 ± 0.6	3.0 ± 0.3	0.4 ± 0.01	175.3 ± 12.3	-	-	12.1 ± 0.9	9.4 ± 0.8	20.6 ± 1.5	227.5		227.4
Uronic acid*	-	-	-	-	37.5 ± 1.3		-	-	-		37.5	
Alditol acetate + uronic acid**	6.7 ± 0.6^a^	3.0 ± 0.3^a^	0.4 ± 0.01	175.3 ± 12.3^a^	37.5 ± 1.3		12.1 ± 0.9^a^	9.4 ± 0.8^a^	20.6 ± 1.5	227.5^a^	37.5^b^	264.9^a^
Carbodiimide	9.0 ± 0.5^b^	4.0 ± 0.5^a^	0.5 ± 0.01	190.1 ± 10.2^c^	1.8 ± 0.1	16.4 ± 0.9	13.0 ± 1.0^a^	12.0 ± 0.8^b^	22.5 ± 1.8	251.1^b^	18.2^a^	269.3^a^
TMS	9.3 ± 0.6^b^	4.2 ± 0.4^a^	0.4 ± 0.01	197.6 ± 7.9^c^	2.0 ± 0.2	18.1 ± 1.1	13.2 ± 0.9^a^	10.7 ± 0.6^a^	20.9 ± 1.3	256.3^b^	20.1^a^	276.4^b^
HPAEC	11.8 ± 0.8^c^	5.0 ± 0.6^b^	0.5 ± 0.01	185.9 ± 8.5^b^	2.9 ± 0.2	17.9 ± 1.3	14.6 ± 0.8^b^	12.3 ± 0.9^b^	21.3 ± 1.9	251.4^b^	20.8^a^	272.2^b^
Rice stem												
Alditol acetate	38.9 ± 3.1	3.0 ± 0.3	1.0 ± 0.02	223.6 ± 9.9	-	-	2.0 ± 0.2	15.1 ± 1.3	29.3 ± 3.1	312.9		312.9
Uronic acid*	-	-	-	-	11.9 ± 0.8		-	-	-		11.9	
Alditol acetate + uronic acid**	38.9 ± 3.1^b^	3.0 ± 0.3^b^	1.0 ± 0.02^b^	223.6 ± 9.9^a^	11.9 ± 0.8		2.0 ± 0.2	15.1 ± 1.3	29.3 ± 3.1	312.9^a^	11.9^b^	324.8^a^
Carbodiimide	41.3 ± 3.8^b^	2.3 ± 0.3^b^	0.4 ± 0.01^a^	225.6 ± 16.2^b^	0.9 ± 0.01	8.0 ± 0.6^b^	2.0 ± 0.2	16.8 ± 0.9	31.3 ± 2.9	319.7^b^	8.9^a^	328.6^a^
TMS	39.5 ± 2.7^b^	2.0 ± 0.2^a^	0.8 ± 0.01^b^	230.3 ± 10.3^b^	1.0 ± 0.01	8.9 ± 0.5^b^	1.5 ± 0.1	15.8 ± 1.4	30.8 ± 2.6	320.7^b^	9.9^a^	330.6^a^
HPAEC	35.9 ± 3.4^a^	2.5 ± 0.2^b^	0.8 ± 0.01^b^	242.7 ± 14.7^c^	1.9 ± 0.02	6.5 ± 0.5^a^	2.9 ± 0.3	14.6 ± 0.7	32.8 ± 2.2	332.2^c^	8.4^a^	340.5^b^
Switchgrass tiller												
Alditol acetate	52.0 ± 2.1	3.3 ± 0.4	0.7 ± 0.01	265.2 ± 9.6	-	-	1.8 ± 0.02	16.5 ± 2.0	22.5 ± 2.1	362.0		361.9
Uronic acid*	-	-	-	-	4.7 ± 0.4		-	-	-		4.7	
Alditol acetate + uronic acid**	52.0 ± 2.1	3.3 ± 0.4^c^	0.7 ± 0.01	265.2 ± 9.6^a^	4.7 ± 0.4		1.8 ± 0.02^b^	16.5 ± 2.0^b^	22.5 ± 2.1	362.0^a^	4.7	366.6^a^
Carbodiimide	50.3 ± 2.6	2.3 ± 0.2^b^	0.4 ± 0.01	270.4 ± 12.7^b^	0.7 ± 0.01	3.0 ± 0.4^a^	1.4 ± 0.02^a^	14.3 ± 0.8^b^	22.4 ± ± 1.9	361.5^a^	3.7	365.1^a^
TMS	52.7 ± 3.2	2.6 ± 0.2^b^	0.3 ± 0.01	278.8 ± ± 17.3^c^	0.8 ± 0.01	3.4 ± 0.4^b^	1.0 ± ± 0.01^a^	13.1 ± 1.1^a^	22.0 ± 2.3	370.5^b^	4.2	374.7^b^
HPAEC	53.6 ± 3.7	1.5 ± 0.1^a^	0.5 ± 0.01	270.6 ± 14.3^b^	0.9 ± 0.01	2.9 ± 0.2^a^	2.1 ± 0.01^c^	15.9 ± 0.9^b^	20.9 ± 2.6	365.1^a^	3.8	368.9^a^

Data are means of three biological replicates ± standard deviation, with two technical replicates for alditol acetate, carbodiimide, and TMS methods (*n* = 6), three technical replicates for uronic acid assay (*n* = 9), and a single technical replicate for HPAEC method (*n* = 3). Different letters in superscript following the values indicate significant differences between the amounts (“a” represents the lowest amount) of a particular (or total) sugar residue in the cell walls of a species across different methods (and not between different types of sugars within that biomass sample analyzed using a particular method). Statistics are one-way analysis of variance (ANOVA) followed by Tukey’s multiple comparison tests with significant *P* value <0.05

* Uronic acid assay data shown here are from analysis using TFA hydrolysis

** Alditol acetate + uronic acid data shown here are the combined data of the respective individual assays

### Glycosyl residue composition analysis by the alditol acetate (AA) method

The glycosyl residue compositions of AIR from leaves of Arabidopsis, *Populus,* rice, and switchgrass were measured by production of AA derivatives followed by GC–MS. The method allows the detection of neutral sugars, but not acidic sugars. Using the AA derivatization method, Gal (28 mol%), Xyl (22 mol%), and Ara (18 mol%) were identified as the predominant non-cellulosic sugars in Arabidopsis leaf cell walls (Fig. 2; Additional file 5). Arabidopsis AIR also had substantial amounts of Rha (12 mol%) and Glc (12 mol%), and lesser amounts of Man (5 mol%) and Fuc (3 mol%). The major sugars in *Populus* leaf AIR were Ara (36 mol%), Xyl (25 mol%), and Gal (18 mol%). These leaf AIR sugar compositions of Arabidopsis and *Populus* with large amounts of Gal and Ara (predominant sugars in pectin) are consistent with the dicot pectin-rich, Type I primary walls [6]. The high level of Xyl is likely from xyloglucan and, to a lesser extent, xylan [6, 51]. In contrast, rice, switchgrass, and other Poales and commelinid monocots have Type II walls that contain arabinoxylans and β1,3:β1,4 mixed-linkage glucans as the predominant hemicellulosic polysaccharides, and have significantly less pectin [46, 52, 53]. This was confirmed by the AA analysis data (Fig. 2; Additional file 5), which identified Xyl, Ara, and Glc as the predominant sugars in leaf AIR from rice (68, 18, and 8 mol%, respectively) and switchgrass (55, 20, and 19 mol%, respectively), as well as significantly lower amounts of Rha. Leaf walls of both grasses had similar relative amounts of the different sugars, with Xyl as the main non-cellulosic sugar. The majority of Xyl in grass walls arises from arabinoxylan, with a smaller amount from xyloglucan [13, 54]. Since the contribution of Ara from pectin is very small in grasses, the Ara was derived largely from arabinoxylan [54].

### Measurement of uronic acid (UA) content of plant biomass

Since the AA method detects only neutral sugars, it was necessary to use an independent method to quantify the amount of acidic sugars in the AIR samples. We analyzed the total UA content of AIR from leaves of Arabidopsis, *Populus*, rice, and switchgrass using a method that combines sulfamate and biphenyl reagents to yield a pink-colored product representative of the UA content. The simultaneous use of sulfamate and biphenyl reagent reduces the brown color, which can develop from neutral sugars and interfere with detection of UAs [37, 38, 55]. Since the hydrolysis procedure used to release monosaccharides from the polymers can affect total sugar yield, here we compared two different hydrolysis methods for the UA analyses. Figure 3A shows that with sulfuric acid hydrolysis, the UA content was 160 and 124 µg/mg for leaf AIR from Arabidopsis and *Populus,* respectively, consistent with dicot Type I primary cell walls that are relatively rich in GalA-containing pectin and in agreement with previously published UA content of AIR from Arabidopsis leaves [56]. As expected for low-pectin-content grass cell walls, the UA content of rice and switchgrass leaf AIR hydrolyzed with sulfuric acid was significantly lower than in the dicots, being 15 and 5 μg/mg AIR, respectively. Compared to sulfuric acid hydrolysis, the TFA hydrolysis yielded lower UA content for all four species, being 109, 91, 12, and 4.5 μg/mg for Arabidopsis, *Populus,* rice, and switchgrass leaf AIR, respectively (Fig. 3B).

### Glycosyl residue composition analysis by the carbodiimide method

Both neutral and acidic sugars can be detected by the carbodiimide method. The carboxylic acid moieties of UAs are activated by a water-soluble carbodiimide to form products that can be reduced to primary alcohols [41] (Fig. 1B). Using this method, GalA (30 mol%), Gal (18 mol%), and Xyl (15 mol%) were the major non-cellulosic monosaccharides detected in Arabidopsis leaf (Fig. 2; Additional file 5), with moderate amounts of Ara (11 mol%), Glc (11 mol%), and Rha (10 mol%). In *Populus* leaf AIR, Ara (38 mol%), Xyl (21 mol%), and Gal (15 mol%) were the major monosaccharides (Fig. 2; Additional file 5), followed by GalA (10 mol%) and Glc (8 mol%). Similar to the trends observed in the AA data above, the carbodiimide method also detected Xyl, Ara, and Glc as the predominant sugars in leaf AIR of rice (66, 19, and 9 mol%, respectively) and switchgrass (58, 20, and 18 mol%, respectively) (Fig. 2; Additional file 5).

### Glycosyl residue composition analysis by the trimethylsilyl (TMS) method

In the TMS method, leaf AIR is hydrolyzed in the presence of methanol to generate methyl glycosides, which are subsequently derivatized with TMS and the resulting TMS ethers separated and identified by GC–MS (Fig. 1C). The TMS analysis identified the most abundant sugar in Arabidopsis leaf AIR as GalA (33 mol%), other major sugars being Gal (16 mol%), Xyl (14 mol%), and lesser amounts of Ara (11 mol%), Glc (10 mol %), Rha (9 mol%), Man (3 mol%), Fuc (2 mol%), and GlcA (1 mol%) (Fig. 2; Additional file 5). The most abundant monosaccharide in *Populus* leaf AIR was Ara (39 mol%) with other major sugars being Xyl (20 mol%), Gal (14 mol%), GalA (11 mol%), and Glc (7 mol%) (Fig. 2; Additional file 5). TMS analysis of rice leaf AIR identified more than 60% of total sugar content as Xyl (66 mol%) and Ara (19 mol%), consistent with the high arabinoxylan content of grass Type II cell walls (Fig. 2; Additional file 5). A considerable amount of Glc (9 mol%) was also present in rice leaf AIR along with measurable amounts of the UAs GalA and GlcA. In switchgrass, Xyl was the most abundant (56 mol%) sugar followed by Ara (22 mol%) and Glc (19 mol%) (Fig. 2; Additional file 5). A smaller amount of Gal (2 mol%) and trace amounts of GalA and GlcA were also detected in switchgrass leaf AIR.

### Glycosyl residue composition analysis by the HPAEC method

Leaf AIR from Arabidopsis, *Populus*, rice, and switchgrass was hydrolyzed with TFA and the resulting monosaccharides were separated and quantified by HPAEC. HPAEC composition analysis of Arabidopsis leaf AIR detected GalA (26 mol%), Gal (19 mol%), and Xyl (18 mol%) as the predominant non-cellulosic cell wall sugars (Fig. 2; Additional file 5). Trace amounts of GlcA and Fuc were also detected. HPAEC analysis of *Populus* leaf AIR indicated a large Ara content (34 mol%) with other major sugars being Xyl (26 mol%) and Gal (16 mol%). Measurable amounts of GalA (8 mol%) and Rha (4 mol%) were also present. The HPAEC data for rice and switchgrass leaf AIR (Fig. 2; Additional files 5) revealed Xyl, Ara, and Glc as the predominant sugars (70, 17, and 8 mol%, respectively, in rice; 60, 20, and 17 mol%, respectively, in switchgrass).

### Comparison of the glycosyl residue composition analysis methods for analysis of leaf biomass

A comparison of the glycosyl residue compositions obtained from leaf AIR from the four different plant sources using the four different analysis methods can be made based on both the relative mol% yield (Fig. 2; Additional file 5) and the μg sugar/mg AIR mass yield (Table 1; Figs. 3, 4) of sugars. An overview of the data showed that the carbodiimide, TMS, and HPAEC methods were able to detect the most common nine neutral and acidic sugars, while the AA method detected the most common seven neutral sugars.

For a more in depth analysis, we first compared the four methods for their ability to detect neutral sugars. All four methods gave the same relative abundance order for the neutral sugars present in leaf AIR from each of the four plant species, based on both the relative (mol%) and the mass (μg/mg AIR) sugar yields (Figs. 2, 4; Table 1; Additional file 5). A minor exception was the HPAEC method which gave reversed orders, compared to the other three methods, for the three least abundant neutral sugars (Rha, Man, and Fuc) in the majority of the samples (Table 1). It is noteworthy, however, that the alditol acetate method often gave the lowest neutral sugar measurement, especially in the dicot samples, as apparent from the total neutral sugar mass yields and from some of the individual sugar (particularly Ara, Xyl, Glc, and Rha) mass yields (Fig. 4; Table 1).

The greatest mass yield of total acidic sugars from leaf AIR samples was obtained using the UA method for Arabidopsis, *Populus,* and rice, and with the TMS method for switchgrass (Table 1). However, the TMS method provided the greatest μg/mg yield of specific acidic sugars (i.e., GlcA and GalA) for all leaf samples (Table 1). The greatest total sugar yield (neutral + acidic sugars) from leaf AIR was obtained using the TMS method for Arabidopsis, *Populus* and switchgrass, and using the HPAEC method for rice (Table 1). Interestingly, the total sugar yield measured from the same amount of starting AIR was much greater from both monocot grasses (~1.3–3.5 times greater) than from the dicots, regardless of the analysis method used (Table 1).

### Comparison of the glycosyl residue composition analysis methods for analysis of wood and stem biomass

To evaluate the efficacy of the four methods for analysis of the sugar composition of biomass biofuel feedstock rich in secondary walls (e.g., stems), we analyzed AIR from *Populus* wood, rice stems, and switchgrass tillers (Table 2; Figs. 5, 6, 7; Additional file 6). The results showed several trends similar to those obtained with the leaf samples. For example, (1) all four methods gave the same relative abundance order of the different neutral sugars based on both mol% and mass yield (μg/mg AIR), again with the exception of reversed orders of Man and Rha abundance using the HPAEC method. (2) As observed in the analysis of leaf AIR, the AA method provided the lowest total mass yield of neutral sugars in the dicot tissue sample, *Populus* wood, compared to the other three methods (Table 2). This trend, however, was again not so obvious for the grass biomass. (3) The greatest amount of total acidic sugars from all feedstock AIR samples was obtained using the UA assay. (4) The total sugar yield from the grass tissues (319–340 and 362–375 µg/mg AIR from rice stem and switchgrass tiller, respectively) was greater than from than from the dicot *Populus* wood (227–276 µg/mg AIR), again a trend similar to that obtained with the leaf samples.

As expected for dicot secondary wall-enriched samples, the Xyl content of *Populus* wood (Fig. 7) was substantially greater than from *Populus* leaves (Fig. 4), due to the abundance of xylan in secondary walls. In contrast, the most measurable change observed in the grass stem and tiller samples (Fig. 7) was a marked decrease in the Ara content compared to the leaf samples (Fig. 4).

## Discussion

Plant cell walls comprise the bulk of plant biomass. The demand for biofuels and bioproducts has spurred research to identify biomass sources with desirable properties and to improve the quality and/or quantity of such biomass. Such studies require comparison of the sugar composition of biomass from different species and from different tissues. Although most biomass feedstocks consist predominantly of cellulose, xylan, and lignin, increasing evidence shows that even seemingly minor components of the biomass (e.g., pectin) can significantly impact wall structure, plant growth, yield, and biomass recalcitrance [15, 21, 22, 57]. Thus, sensitive, accurate, and preferably high-throughput analytical method(s) are needed to identify and quantify the different major and minor neutral and acidic sugars that constitute the non-cellulosic polysaccharides of plant cell walls.

Here we assessed four different methods, i.e., AA–UA assay, carbodiimide, TMS, and HPAEC, for their ability to quantitatively measure the sugar composition of non-cellulosic polysaccharides in cell walls from four different species representing dicots and grasses. Since the hydrolysis conditions used in these methods do not appreciably hydrolyze cellulose, the results are indicative of non-cellulosic sugar content. The four methods were compared for their ability to detect and quantify the nine most common monosaccharides present in plant cell walls, the yield of sugar detected by each method, and the ease and practicality of use of each method (summarized in Additional file 7).

All four methods were able to detect and quantify the seven major neutral sugars in both leaf and stem biomass samples from the different species, even at relatively low amounts. All four methods also gave the same mol% and µg sugar/mg AIR abundance ranking of the neutral sugars, with a minor exception of the HPAEC method for which the abundance ranking of the less abundant sugars Rha, Man, and Fuc was often reversed compared to the other methods. However, only three of the methods, the carbodiimide, TMS and HPAEC, were able provide a comparable quantitative and qualitative evaluation of the nine major neutral and acidic sugars present in all plant biomass.

The AA method is the most commonly used method for sugar composition analysis of plant biomass, likely due to the relatively simple GC chromatograms produced which have single peaks for each sugar, making quantification easier [12]. However, the inability of the AA method to provide quantitative data for the specific acidic sugars (i.e., GalA and GlcA) is a major limitation when measuring the composition of plant cell wall biomass, since these sugars are critical components in the pectic and hemicellulosic polymers. The UA assay is often carried out in conjunction with the AA method to complement the results of the AA method and provide a measure of the total acidic sugar content of the tissue. However, the UA assay does not provide information about the amounts of the individual acidic sugars, GlcA and GalA. In this study, we compared the use of TFA, a solvent used in the AA method, versus the more typical sulfuric acid, to hydrolyze AIR samples for the UA analysis. The yield of UA was lower using TFA hydrolysis compared to hydrolysis by sulfuric acid [11, 58, 59]. However, even with TFA hydrolysis, the UA assay still generally provided the greatest total UA values compared to the other three methods, particularly from samples with high pectin content such as in the dicot samples (Tables 1, 2). For example, inspection of µg sugar/mg AIR data for Arabidopsis leaf (Table 1) indicates that acidic sugars (GlcA + GalA) account for 29, 32, and 29% of the biomass based on analyses using the carbodiimide, TMS and HPAEC methods, respectively, but rather 47% of the biomass based on AA and UA assays. Thus, the comparative results presented here show that the amount of total UAs measured using the AA–UA methods may not be comparable to the amount of GalA + GlcA detected using the carbodiimide, TMS and HPAEC methods. Furthermore, although the acidic sugar yield was high using the UA assay, it does not differentiate between GalA and GlcA which is necessary to study specific wall components such as pectin and glucuronoxylan, respectively. Thus, the AA–UA assay method does not provide complete sugar composition information for plant biomass, and it yields a different relative amount of acidic versus neutral sugars compared to the other three methods.

The carbodiimide method takes advantage of the simplicity of AA chromatographic profiles by reducing the UAs to their neutral sugars prior to the AA procedure, thus enabling detection of GalA and GlcA in addition to the neutral sugars. Its drawbacks, however, include the time-consuming and laborious steps required to modify the UAs, which added up to three additional days of experimental time on top of that needed for the AA part of the procedure, and the greater amount of starting AIR needed (e.g., 10 mg versus 100–400 µg) (see “Methods”; Additional file 7). Moreover, to quantify the UAs, the amounts of GalA and GlcA are indirectly determined by comparing the Gal and Glc peaks obtained from the AA and the carbodiimide methods [42], requiring a sample to be measured in parallel by both methods. Thus, twice the number of samples need to be processed (compared to the AA method) when using the carbodiimide method.

The TMS method requires the simplest sample preparation compared to the other GC–MS-based methods. In our hands, this method yielded the highest overall amounts of sugar for Arabidopsis, *Populus* and switchgrass and comparable amounts for rice, compared to the other methods. It also detected the greatest amount of GalA and GlcA in AIR samples from most samples compared to the carbodiimide and HPAEC methods. The major difficulty with the TMS method is the interpretation of the GC profiles. TMS derivatization of methyl glycosides results in derivatives of both the α- and β-anomeric configurations as well as the pyranose and furanose ring forms of each sugar, yielding multiple peaks for each sugar in the chromatogram. This can be managed, however, by comparison of the sample chromatograms with chromatograms of respective sugar standards and confirmation of peak identity from the MS spectra. Beyond routine plant cell wall sugar composition analysis, the TMS method also allows detection of amino sugars [60], other unusual sugars (e.g., 2-*O*-methylxylose, 2-*O*-methylfucose, acetic acid, Kdo, Dha) [20], and fatty acids [61], making it a versatile analytical method.

In the HPAEC method, hydrolyzed sugars are analyzed directly by liquid chromatography with electrochemical detection, without the need for a time-consuming and sometimes incomplete derivatization step. The HPAEC method clearly required the least amount of time for sample preparation compared to the other methods. For this study, we chose to perform the chromatography using two different columns/gradients to enable accurate detection and quantification of all nine monosaccharides, with the downside that a longer analysis time was required per sample. Other HPAEC gradient schemes that allowed separation of the nine sugars using one column in a single run have been reported [31, 45], which may reduce the analysis time considerably. However, in our hands these methods did not provide sufficient base line separation for Xyl and Man, especially for cell wall samples that are rich in xylan and/or xyloglucan. Another drawback of the HPAEC method is that it is not readily compatible with MS to allow confirmation of peak identity, a critical limitation since HPAEC retention time alone is (sometimes) not sufficient to conclusively identify a compound. The sugar peaks could indeed be collected, but would require further treatment, e.g., to remove salts and reduce sample volumes before being subjected for MS verification. Such steps would add labor and time factors to the analysis.

With the above differences noted, all four methods tested enabled general conclusions regarding the cell wall content of the biomass to be made. For example, all four methods indicated significant differences in the glycosyl residue composition of leaf AIR from the grasses switchgrass and rice (Type II cell walls) compared to the dicots Arabidopsis and *Populus* (Type I cell walls). The latter were relatively richer in UAs, particularly GalA, which is consistent with the higher pectin content and the former were richer in Xyl and Glc, which is consistent with the greater xylan content and presence of mixed-linkage glucans in grass primary walls. The analysis of *Populus* wood, rice stem, and switchgrass tiller using the four methods provided sugar composition results consistent with tissues enriched in secondary walls (Table 2; Figs. 5, 6; Additional file 6). For example, these methods identified a greater amount of Xyl and a reduced amount of GalA, Rha, Ara, and Gal in *Populus* wood (Table 2) compared to *Populus* leaf (Table 1), consistent with the higher glucuronoxylan and lower pectin content in *Populus* secondary walls compared to primary walls. The results also yielded some unexpected findings. Overall, all four methods detected a greater amount of total sugar from the same amount of starting AIR from grasses compared to dicots, regardless of whether the tissues were enriched in primary or secondary walls (compare total sugar values in Tables 1, 2). This result suggests that the non-cellulosic polysaccharides may be present in greater amounts in grasses than in dicots [46]. Alternatively, it is possible that the non-cellulosic polysaccharides are held less tightly in the walls of grasses than in dicots. For example, intrinsic differences in the cell wall structure and/or architecture of these two different phylogenetic groups of plants, such as distinct cross-linking between wall components and different overall wall structural features, could account for the observation. This phenomenon warrants further study.

Although all four methods provided generally comparable sugar compositions, the results indicate that use of the AA + UA method alone to analyze plant biomass has limitations compared to the other methods. The results also make clear that the choice of glycosyl residue composition analysis method is critical to obtain a complete set of neutral + acidic sugar composition data for the analysis of cell wall polymers in biomass and for detailed mechanistic interpretation of the results. According to the American Society for Testing and Materials [9], the AA and TMS methods are the most accurate for analysis of sugars in plant biomass. Based on our comparison, we found that the TMS method gave a slightly greater yield of the majority of sugars, including acidic sugars, in plant biomass (Tables 1, 2), although the carbodiimide and HPAEC methods also provided highly comparable results. In summary, this study provides a basis for selecting a sugar analysis method that is commensurate with the experimental goals. We recommend that the TMS, HPAEC, or carbodiimide methods be used when the goals include detailed mechanistic interpretations regarding plant cell wall (biomass) structure.

## Additional files

**Additional file 1.** Gas chromatographic (GC) profile of the derivatized sugar standards in the alditol acetate (AA) method. The standard mixture consists of 0.5 μg of each sugar (in bold), supplemented with myo-inositol (0.2 μg, in bold) as an internal standard. Note that ribose (in brackets) is also included in the chromatogram shown. Derivatized sugars are separated on a SP-2330 Supelco column (30 m × 0.25 mm, 0.25 μm film thickness) connected to a Hewlett–Packard chromatograph (5890) using helium as the carrier gas with an oven temperature program as described in the “Methods”.

**Additional file 2.** An example of the standard curve used in the uronic acid assay.

**Additional file 3.** Gas chromatographic (GC) profiles of the derivatized sugar standards in the trimethylsilyl (TMS) method. The standard mixtures 1 and 2 consist of the nine monosaccharides (each 0.5 μg, shown in bold): arabinose (Ara), rhamnose (Rha), fucose (Fuc), xylose (Xyl), mannose (Man), galactose (Gal), glucose (Glc), galacturonic acid (GalA), and glucuronic acid (GlcA), supplemented with myo-inositol (Inos, 0.2 μg, in bold) as an internal standard. Also included in the chromatogram shown are (in parentheses) ribose (Rib), *N*-acetylmannosamine (ManNac), *N*-acetylglucosamine (GlcNAc), and *N*-acetylgalactosamine (GalNAc). The derivatized sugars are separated on a Supelco EC-1 fused silica capillary column (30 m × 0.25 mm ID) on an Agilent 7890A gas chromatograph using helium as the carrier gas with temperature gradient as described in the “Methods”.

**Additional file 4.** Chromatographic profiles of the sugar standards in the HPAEC method. As outlined in “Methods” section, the HPAEC analyses were carried out in two separate runs using two different programs (i.e. different columns and gradients) for each sample. In bold are the sugars quantified using the respective program. (A) Program 1 was used to quantify the amounts of fucose (Fuc), rhamnose (Rha), arabinose (Ara), galactose (Gal), glucose (Glc), galacturonic acid (GalA), and glucuronic acid (GlcA) on a Dionex PA20 column eluted using a NaOH/NaOAc gradient. (B) Program 2 was used to quantify the amounts of xylose (Xyl) and mannose (Man), which eluted as one peak in program 1, on a Dionex PA1 column eluted isocratically using 2 mM NaOH.

**Additional file 5.** Comparison of the mol% of the different types of sugars in leaf AIR from Arabidopsis, *Populus*, rice and switchgrass using the four different glycosyl residue composition analysis methods.

**Additional file 6.** Comparison of the mol% of the different types of sugars in AIR from *Populus* wood, rice stem and switchgrass tiller biomass using the four different glycosyl residue composition analysis methods.

**Additional file 7.** Comparison of the four glycosyl residue composition analysis methods.

## Abbreviations

AA: alditol acetate
AIR: alcohol insoluble residue
Ara: arabinose
ddH_2_O: deionized water
ECD: electrochemical detection
Fuc: fucose
Gal: galactose
GalA: galacturonic acid
GC–MS: gas chromatography–mass spectrometry
Glc: glucose
GlcA: glucuronic acid
HPAEC: high-pressure, anion-exchange chromatography
Man: mannose
Rha: rhamnose
RT: room temperature
TFA: trifluoroacetic acid
TMS: trimethylsilyl
UA: uronic acid
Xyl: xylose
WT: wild type
