# Supplementary material for: Comparison of four glycosyl residue composition methods for effectiveness in detecting sugars from cell walls of dicot and grass tissues
Source: Biotechnol Biofuels. 2017 Jul 14;10:182. doi: 10.1186/s13068-017-0866-1 (PMC5513058; doi:10.1186/s13068-017-0866-1)
Supplement: Supplementary file 1 — Additional file 1. Gas chromatographic (GC) profile of the derivatized sugar standards in the alditol acetate (AA) method. The standard mixture consists of 0.5 μg of each sugar (in bold), supplemented with myo-inositol (0.2 μg, in bold) as an internal standard. Note that ribose (in brackets) is also included in the chromatogram shown. Derivatized sugars are separated on a SP-2330 Supelco column (30 m × 0.25 mm, 0.25 μm film thickness) connected to a Hewlett–Packard chromatograph (5890) using helium as the carrier gas with an oven temperature program as described in the “Methods”. [file 13068_2017_866_MOESM1_ESM.docx]

**Additional file 1** – Gas chromatographic (GC) profile of the derivatized sugar standards in the alditol acetate (AA) method. The standard mixture consists of 0.5 μg of each sugar (in bold), supplemented with myo-inositol (0.2 μg, in bold) as an internal standard. Note that ribose (in brackets) is also included in the chromatogram shown. Derivatized sugars are separated on a SP-2330 Supelco column (30 m x 0.25 mm, 0.25 μm film thickness) connected to a Hewlett–Packard chromatograph (5890) using helium as the carrier gas with an oven temperature program as described in the Methods.
