# Supplementary material for: Comparison of four glycosyl residue composition methods for effectiveness in detecting sugars from cell walls of dicot and grass tissues
Source: Biotechnol Biofuels. 2017 Jul 14;10:182. doi: 10.1186/s13068-017-0866-1 (PMC5513058; doi:10.1186/s13068-017-0866-1)
Supplement: Supplementary file 2 — Additional file 2. An example of the standard curve used in the uronic acid assay. [file 13068_2017_866_MOESM2_ESM.docx]

**Additional file 2** – An example of the standard curve used in the uronic acid assay.
