# Supplementary material for: Comparison of four glycosyl residue composition methods for effectiveness in detecting sugars from cell walls of dicot and grass tissues
Source: Biotechnol Biofuels. 2017 Jul 14;10:182. doi: 10.1186/s13068-017-0866-1 (PMC5513058; doi:10.1186/s13068-017-0866-1)
Supplement: Supplementary file 3 — Additional file 3. Gas chromatographic (GC) profiles of the derivatized sugar standards in the trimethylsilyl (TMS) method. The standard mixtures 1 and 2 consist of the nine monosaccharides (each 0.5 μg, shown in bold): arabinose (Ara), rhamnose (Rha), fucose (Fuc), xylose (Xyl), mannose (Man), galactose (Gal), glucose (Glc), galacturonic acid (GalA), and glucuronic acid (GlcA), supplemented with myo-inositol (Inos, 0.2 μg, in bold) as an internal standard. Also included in the chromatogram shown are (in parentheses) ribose (Rib), N-acetylmannosamine (ManNac), N-acetylglucosamine (GlcNAc), and N-acetylgalactosamine (GalNAc). The derivatized sugars are separated on a Supelco EC-1 fused silica capillary column (30 m × 0.25 mm ID) on an Agilent 7890A gas chromatograph using helium as the carrier gas with temperature gradient as described in the “Methods”. [file 13068_2017_866_MOESM3_ESM.docx]

**Additional file 3** – Gas chromatographic (GC) profiles of the derivatized sugar standards in the trimethylsilyl (TMS) method.

The standard mixtures 1 and 2 consist of the nine monosaccharides (each 0.5 μg, shown in bold): arabinose (Ara), rhamnose (Rha), fucose (Fuc), xylose (Xyl), mannose (Man), galactose (Gal), glucose (Glc), galacturonic acid (GalA), and glucuronic acid (GlcA), supplemented with myo-inositol (Inos, 0.2 μg, in bold) as an internal standard. Also included in the chromatogram shown are (in parentheses) ribose (Rib), *N*-acetylmannosamine (ManNac), *N*-acetylglucosamine (GlcNAc), and *N*-acetylgalactosamine (GalNAc). The derivatized sugars are separated on a Supelco EC-1 fused silica capillary column (30m × 0.25 mm ID) on an Agilent 7890A gas chromatograph using helium as the carrier gas with temperature gradient as described in the Methods.
