# Supplementary material for: Comparison of four glycosyl residue composition methods for effectiveness in detecting sugars from cell walls of dicot and grass tissues
Source: Biotechnol Biofuels. 2017 Jul 14;10:182. doi: 10.1186/s13068-017-0866-1 (PMC5513058; doi:10.1186/s13068-017-0866-1)
Supplement: Supplementary file 4 — Additional file 4. Chromatographic profiles of the sugar standards in the HPAEC method. As outlined in “Methods” section, the HPAEC analyses were carried out in two separate runs using two different programs (i.e. different columns and gradients) for each sample. In bold are the sugars quantified using the respective program. (A) Program 1 was used to quantify the amounts of fucose (Fuc), rhamnose (Rha), arabinose (Ara), galactose (Gal), glucose (Glc), galacturonic acid (GalA), and glucuronic acid (GlcA) on a Dionex PA20 column eluted using a NaOH/NaOAc gradient. (B) Program 2 was used to quantify the amounts of xylose (Xyl) and mannose (Man), which eluted as one peak in program 1, on a Dionex PA1 column eluted isocratically using 2 mM NaOH. [file 13068_2017_866_MOESM4_ESM.docx]

**Additional file 4** – Chromatographic profiles of the sugar standards in the HPAEC method. As outlined in the Methods section, the HPAEC analyses were carried out in two separate runs using two different programs (i.e. different columns and gradients) for each sample. In bold are the sugars quantified using the respective program. (A) Program 1 was used to quantify the amounts of fucose (Fuc), rhamnose (Rha), arabinose (Ara), galactose (Gal), glucose (Glc), galacturonic acid (GalA), and glucuronic acid (GlcA) on a Dionex PA20 column eluted using a NaOH/NaOAc gradient. (B) Program 2 was used to quantify the amounts of xylose (Xyl) and mannose (Man), which eluted as one peak in program 1, on a Dionex PA1 column eluted isocratically using 2 mM NaOH.
