# Supplementary material for: Comparison of four glycosyl residue composition methods for effectiveness in detecting sugars from cell walls of dicot and grass tissues
Source: Biotechnol Biofuels. 2017 Jul 14;10:182. doi: 10.1186/s13068-017-0866-1 (PMC5513058; doi:10.1186/s13068-017-0866-1)
Supplement: Supplementary file 7 — Additional file 7. Comparison of the four glycosyl residue composition analysis methods. [file 13068_2017_866_MOESM7_ESM.docx]

**Additional file 7** - Comparison of the four glycosyl residue composition analysis methods.

| **Method features** | **Alditol acetate + uronic acid** | | **Carbodiimide** | **TMS** | **HPAEC** |
| --- | --- | --- | --- | --- | --- |
|  | **Alditol acetate** | **Uronic acid** |  |  |  |
| **Starting sample** | AIR  (or AIR fractions) | AIR  (or AIR fractions) | AIR  (or AIR fractions) | AIR  (or AIR fractions) | AIR  (or AIR fractions) |
| **Amount of sample needed** | 100-500 μg | 400-800 μg | 10 mg | 100-500 μg | 100 μg |
| **Preparation of sample for analysis** | - Hydrolysis - Reduction - *O*-acetylation | - Hydrolysis - *m*-hydroxybiphenyl assay | - Uronic acid activation - 1^st^ reduction - Hydrolysis - 2^nd^ reduction - *O*-acetylation | - Methanolysis - TMS derivatization | - Hydrolysis |
| **Hydrolysis conditions** | 2 M TFA,121°C, 2 h | - 12.5 mM Na_2_B_4_O_7_ in concentrated H_2_SO_4_, 100°C, 20 min,   -or-   - 2M TFA,120°C, 2h | 2 M TFA, 121°C, 2h | 1 M methanolic-HCl, 80ºC, 18 h | 2 M TFA, 120°C, 1 h |
| **Analytical method and running time / sample** | GC-MS  45 min. | Colorimetry  5-10 min. | GC-MS  45 min. | GC-MS  45 min. | HPAEC  Program1 – 35 min.  Program2 – 40 min. |
| **Data obtained and analysis** | - GC peaks identified and quantified by comparison to respective sugar and internal standards - MS confirmation of peak identity | - Absorbance at 540 nm used to estimate UA content by comparison to a GalA standard curve | - GC peaks identified and quantified by comparison to respective sugar and internal standards - MS confirmation of peak identity | - GC peaks identified and quantified by comparison to respective sugar and internal standards - MS confirmation of peak identity | - HPAEC peaks identified and quantified by comparison to respective sugar standards. |
| **Glycosyl residue detection** | - Neutral sugar  (Ara, Rha, Fuc, Xyl, Man, Gal, Glc) | - Total uronic acid (GlcA + GalA) | - Ara, Rha, Fuc, Xyl, GlcA, GalA, Man, Gal, Glc | - Ara, Rha, Fuc, Xyl, GlcA, GalA, Man, Gal, Glc | - Ara, Rha, Fuc, Xyl, GlcA, GalA, Man, Gal, Glc |
| **Advantages** | - Derivatized compounds are very stable - Elimination/ modification of anomeric center results in simple, easy to quantify chromatogram with a single peak for each sugar |  | - Can recognize all nine monosaccharides | - Can recognize all nine monosaccharides - Relatively mild hydrolysis condition, results in large amount of hydrolyzed material available for derivatization - Rapid derivatization method proceeds under relatively mild conditions (Ruiz-Matute *et al.*, 2011) | - Can recognize all nine monosaccharides - No derivatization step(s) |
| **Disadvantages** | - Cannot detect acidic (and amino) sugars | - Cannot differentiate specific uronic acids (GalA vs. GlcA) | - Large amount of material needed - Time-consuming and tedious extra uronic acid reduction steps | - Complex chromatogram with multiple peaks for each sugar | - No MS confirmation of peak identity |

**References:**

**Ruiz-Matute AI, Hernandez-Hernandez O, Rodriguez-Sanchez S, Sanz ML, Martinez-Castro I. 2011.** Derivatization of carbohydrates for GC and GC-MS analyses. *J Chromatogr B Analyt Technol Biomed Life Sci* **879**(17-18): 1226-1240.
